# Acoustic ranging in poison frogs—it is not about signal amplitude alone

**DOI:** 10.1007/s00265-017-2340-2

**Published:** 2017-07-12

**Authors:** Max Ringler, Georgine Szipl, Walter Hödl, Leander Khil, Barbara Kofler, Michael Lonauer, Christina Provin, Eva Ringler

**Affiliations:** 1 Department of Ecology and Evolutionary Biology, University of California Los Angeles, 621 Charles E. Young Drive South, Los Angeles, CA 90095-1606 USA; 2 Department of Integrative Zoology, University of Vienna, Althanstrasse 14, 1090 Vienna, Austria; 3 Department of Cognitive Biology, University of Vienna, Althanstrasse 14, 1090 Vienna, Austria; 4 Konrad Lorenz Forschungsstelle, Core Facility, University of Vienna, Fischerau 11, 4645 Grünau im Almtal, Austria; 5 Messerli Research Institute, University of Veterinary Medicine Vienna, Medical University of Vienna, and University of Vienna, Veterinärplatz 1, 1210 Vienna, Austria

**Keywords:** Acoustic ranging, Playback experiment, Territoriality, Poison frogs, Signal-to-noise ratio, SNR, SPL

## Abstract

**Abstract:**

Acoustic ranging allows identifying the distance of a sound source and mediates inter-individual spacing and aggression in territorial species. Birds and mammals are known to use more complex cues than only sound pressure level (SPL), which can be influenced by the signaller and signal transmission in non-predictable ways and thus is not reliable by itself. For frogs, only SPL is currently known to mediate inter-individual distances, but we hypothesise that the strong territoriality of Dendrobatids could make the use of complex cues for ranging highly beneficial for this family. Therefore, we tested the ranging abilities of territorial males of *Allobates femoralis* (Dendrobatidae, Aromobatinae) in playback trials, using amplitude-normalized signals that were naturally degraded over distance, and synthetic signals that were masked with different levels of noise. Frogs responded significantly less to signals recorded from larger distances, regardless of SPL and signal-to-noise ratio (SNR), but showed no differential response to natural minimum and maximum SNRs across the typical communication range in wild populations. This indicates that frogs used signal amplitude and SNR only as ancillary cues when assessing the distance of sound sources and relied instead mainly on more complex cues, such as spectral degradation or reverberation. We suggest that this ability mediates territorial spacing and mate choice in *A. femoralis*. Good ranging abilities might also play a role in the remarkable orientation performance of this species, probably by enabling the establishment of a mental acoustic map of the habitat.

**Significance statement:**

Acoustic ranging allows the distance of vocalizing competitors and mates to be identified. While birds and mammals are known to use complex cues such as temporal degradation, frequency-dependent attenuation and reverberation for ranging, previous research indicated that frogs rely only on signal amplitude (sound pressure level) to assess the distance of other callers. The present study shows for the first time that also poison frogs can make use of more complex cues, an ability which is likely to be highly beneficial in their territorial social organization and probably can also be used for orientation.

**Electronic supplementary material:**

The online version of this article (doi:10.1007/s00265-017-2340-2) contains supplementary material, which is available to authorized users.

## Introduction

The ability to localize the direction and distance of vocalizing conspecifics is generally advantageous for animals that use sound to communicate as it allows early decisions to be made before direct contact occurs and reduces the risk of unnecessary aggressive responses (Erulkar 1972; McGregor 1994; Bradbury and Vehrencamp 2011; Hardy and Briffa 2013; Bee et al. 2016). Together with individual identification and eavesdropping, acoustic distance assessment plays a crucial role in territorial social systems of vocal species (McGregor 1993) and commonly mediates inter-individual spacing by informing territory holders about the proximity and thus the threat potential of nearby callers (Brown and Orians 1970; Robertson 1984; Naguib et al. 2008, 2011). Thus, acoustic territory advertisement and ranging allow animals to avoid more costly physical contests and fights over territories (Whitney and Krebs 1975; Richards 1981; Morton 1986; Bee et al. 2016).

Physically, a sound’s most straightforward distance cue is its amplitude, since it depends directly on the distance from the sound source. Acoustic signal amplitude corresponds to sound pressure, which is the local pressure deviation from the ambient atmospheric pressure caused by a sound event. The sound pressure level (SPL) is usually given in decibel (dB) in a logarithmic relation to 20 μPa (for airborne sound), the threshold of human hearing. Under free spherical, atmospheric spreading, sound pressure follows the inverse distance law by 1/*r* with distance *r* from the sound source, resulting in an SPL drop of −6 dB per doubling of the distance *r* (Rossing 2007; Bradbury and Vehrencamp 2011). Thus, if a receiver knows the original SPL of a sound at its source, or when it moves back and forth in its far field, a receiver should be able to estimate signaller distance from the perceived SPL (Naguib 1997b; Nelson 2000). However, SPL alone is not a reliable cue for distance assessment, as a caller could not only be closer to or further away from the receiver, but could also actively vary call amplitude (Richards 1981; Morton 1982). And once emitted, the signal could undergo unpredictable excess attenuation during transmission, caused by vegetation or ground structures, or distortion by wind and temperature gradients (Erulkar 1972; Morton 1975, 1986; Naguib 1997b; Ellinger and Hödl 2003; Kreutz-Erdtmann and Lima Pimentel 2013).

As an adaptation to this restriction, at least birds and mammals (for humans, see Zahorik et al. (2005)) have evolved the ability to also use several more complex cues that do not follow a simple physical law to assess the distance of a sound source. The ranging hypothesis identifies overall temporal degradation, frequency-specific degradation, frequency-specific attenuation and/or reverberation of a signal as cues for distance assessment and has been mainly tested in birds (Richards and Wiley 1980; Morton 1982, 1986; Dabelsteen et al. 1993; McGregor 1993, 1994; Holland et al. 2001; Naguib and Wiley 2001). These cues are more complex than SPL in that they require the concurrent perception and assessment of temporal, spectral and intensity characteristics of a signal. Furthermore, they are highly habitat dependent and not every cue might be present in every vocalization context (e.g. tonal and atonal call components (Sun et al. 2000; Bernal et al. 2009; Bonachea and Ryan 2011) or tonal advertisement and atonal courtship calls (Weygoldt 1980; Simões et al. 2010; Kollarits et al. 2017)); thus, their correct interpretation for ranging depends on experience with the signal (Morton 1982, 1998); but see Naguib (1997a) for an example of inexperienced ranging and Naguib (1998) and Wiley (1998) for a discussion of the role of experience in ranging).

In frogs, only signal amplitude/SPL has been identified so far as a cue for acoustic distance assessment and spacing. This is the case for several species: Blanchard’s cricket frogs (*Acris crepitans blanchardi*, Wagner Jr. 1989), common tink frogs (*Diasporus* (*Eleutherodactylus*) *diastema*, Wilczynski and Brenowitz 1988), barking treefrogs (*Hyla gratiosa*, Murphy and Floyd 2005), Pacific treefrogs (*Hyla regilla*, (Whitney and Krebs 1975; Brenowitz 1989), gray treefrogs (*Hyla versicolor*, Fellers 1979), painted reedfrogs (*Hyperolius mamoratus*, Telford 1985), strawberry poison frogs (*Oophaga* (*Dendrobates*) *pumilio*, Bunnel 1973), spring peepers (*Pseudacris* (*Hyla*) *crucifer*, Gerhardt et al. 1989) and wrinkled toadlets (*Uperoleia rugosa*, Robertson 1984). In these species, SPL threshold values maintain inter-individual distances in aggregations and elicit aggressive responses, or mediate graded responses which are expressed proportionally to SPL (Velez et al. 2013). So far, only little evidence exists that frogs also use more complex cues for ranging, although the possibility has been discussed previously (Ryan and Sullivan 1989; Murphy 2008). However, owing to their poikilotherm physiology, frogs have a highly sedentary lifestyle (Wells 2007), and therefore, it would presumably be highly beneficial to use all available information to remotely assess competitors to optimize energy expenditure in contests (cf. Dyson et al. 2013; Bee et al. 2016).

The lack of evidence for ranging in anurans may partly be due to a lack of research, as unlike the multitude of studies on directional localization and source segregation in anurans (see Gerhardt and Huber 2002; Christensen-Dalsgaard 2005; Narins et al. 2006; Bee and Christensen-Dalsgaard 2016), so far very few studies have specifically addressed cues used in anuran distance assessment. In playback trials with barking treefrogs (*Hyla gratiosa*), Murphy (2008) did not find evidence for the proposed mechanisms of anuran distance assessment and suggested the use of more complex methods such as triangulation during movement. Using playbacks of normalized, naturally degraded calls of male gray treefrogs (*Hyla versicolor*), Schwartz et al. (2016) demonstrated that females in this species prefer undegraded calls from closer callers, indicating the perception of signal degradation. Venator et al. (2017) found evidence for the use of temporal degradation for distance assessment in addition to signal amplitude in male cricket frogs (*Acris crepitans*); however, they did not rule out concurrent inhibitory effects of signal degradation on call recognition in their study. For the austral forest frogs *Eupsophus emiliopugini* and *E. calcaratus*, Penna et al. (2017) found a strong dependence of vocal responses to stimulus SPL but no difference in the frogs’ calling activity in response to synthetic pulse amplitude modulation of stimuli that were mimicking naturally degraded calls.

Previous research showed that frogs can recognize and interpret temporally degraded signals (Kuczynski et al. 2010; see Göd et al. (2007) and Vélez et al. ( 2012) for *Allobates femoralis*) and that they are capable of long-term integration in their auditory system (Alder and Rose 1998). However, other neurological and cognitive constraints on signal processing (cf. Narins et al. 2006) might still limit ranging mechanisms to simpler cues in frogs. In contrast to most birdsong, the vocalizations of frogs are not learned but inherited and highly stereotypic and receive only little contextual or experience-based modification (Hauser 1996; Narins et al. 2006). This means that frogs come with an innate template of their own calls and probably do not need to gain experience with the calls of conspecifics.

Neotropical poison frogs (Dendrobatidae) are known for their territorial social organization, and males of many species advertise their territories through prolonged calling to repel competitors and attract mates (Pröhl 2005; Lötters et al. 2007). Therefore, good ranging skills, integrating more complex cues than SPL alone to make best use of all available information, would be especially beneficial for frogs of this family, rendering them a promising group to search for such abilities in anurans. We tested the ranging abilities of *A. femoralis* (Dendrobatidae, Aromobatinae) by examining the response of males in playback trials with amplitude-normalized, naturally distance-degraded, conspecific advertisement calls, while controlling for distance and perceived SPL.

In the absence of SPL-related cues, we expected males to still exhibit stronger responses to test signals recorded from closer distances, potentially within an individual’s territory. In turn, responses should be weaker to signals that were recorded across longer distances, likely from outside an individual’s territory (McGregor 1994). The phonotactic responses in *A. femoralis* are hierarchical, consisting of initial head body orientation (HBO), followed by movement towards the potential intruder, and eventually resulting in a full approach. Given proper signal recognition, we expected a wider initial response (HBO) across signaller ranges, as frogs should also pay attention to lesser potential threats, for which subsequent evaluation may lead to no further aggressive response (movement, approach). In turn, under ranging the discrimination between near and far signals in the later response categories ‘movement’ and ‘approach’ should be more pronounced when threats are correctly assessed, as only higher threats require a full aggressive response. To assess the impact of noise and the signal-to-noise ratio (SNR) on the phonotactic responses of *A. femoralis* males, we conducted a follow-up experiment with test signals where we only manipulated the SNR of the test signal to the minimum and maximum SNR found in the experiment with naturally degraded signals. Similar phonotactic responses of the tested frogs to both conditions would be indicative that SNR and noise level in the range of the test signals in the first experiment did not play a role in distance assessment with the naturally degraded signals.

## Materials and methods

### Study species

*Allobates femoralis* is a Neotropical poison frog (Dendrobatidae) from a species complex with a pan-Amazonian distribution (Amézquita et al. 2009; Fouquet et al. 2012). Males are highly territorial throughout the prolonged breeding season (Roithmair 1992; Ringler et al. 2009), when they announce their territories and try to attract females with extensive calling from elevated perches on the forest floor (Narins et al. 2005). The advertisement call, emitted at 92 dB SPL (re 20 μPa) measured at a distance of 50 cm (Hödl 1987), consists of four notes, each sweeping upwards in the frequency range of 2900–3900 Hz ((Narins et al. 2003; Gasser et al. 2009); see insert in Fig. 2). Males call from sunrise until sunset, and calling activity peaks from 1500 to 1600 h and is lowest around 1200 h (Hödl 1983; Kaefer et al. 2012). Possessing a territory is a prerequisite for male reproductive success (Ursprung et al. 2011), and as a consequence, territories are vigorously defended against calling intruders which are approached and attacked immediately (Narins et al. 2003; Ringler et al. 2011). Territory intrusion can be simulated by broadcasting conspecific calls with a loudspeaker, which is immediately approached phonotactically by males in playback trials (Hödl 1983; Ursprung et al. 2009; Ringler et al. 2011). However, to elicit a full physical attack, the additional optical stimulus of the pulsating vocal sac is required, as was demonstrated using robotic model frogs (Narins et al. 2003, 2005). This phonotactic response is only exhibited above a certain SPL threshold, which was found to be 56–68 dB for a head-body-orientation (HBO) and subsequent antiphonal calling towards the source and >68 dB for a phonotactic approach in a Peruvian population of *A. femoralis* (Hödl 1987).

### Study period and site

The playback trials with normalized, naturally degraded signals were conducted between 30 January and 17 February 2015, and the trials testing the effect of SNR were performed between 01 and 08 March 2017. Hence, we conducted this study at the onset of the rainy season, when males were highly territorial and their calling activity was high due to the concurrent breeding season. We performed the playback experiments in an *A. femoralis* population located in an 8.3 ha lowland rainforest plot (Ringler et al. 2015b; Ringler et al. 2016) near the field camp ‘Saut Pararé’ (4°02′ N, 52°41′ W, WGS84) of the CNRS Nouragues Ecological Research Station (http://www.nouragues.cnrs.fr; Bongers et al. 2001).

### Experiment 1—playbacks with degraded signals

#### Test signals

The test signals for the conflicting-properties playbacks to assess the ranging abilities of frogs were obtained during a study on understory sound transmission characteristics (MR et al. unpublished data). We used the original synthetic call by Narins et al. (2003; termed ‘standard call’ by Ursprung et al. (2009)) as the base signal, which was composed from natural recordings to feature the average spectral and temporal call parameters of an *A. femoralis* population near ‘Camp Arataï’ (Gasser et al. 2009), ~35 km downstream from the Pararé population. As such, this call represented a neutral intruding individual for frogs in the Pararé population, unknown to all tested individuals and likely to elicit an equal and reliable aggressive response across all tested individuals. The original recordings for this synthetic call were made with a cassette tape recorder (Professional Walkman WMD6C, Sony, Tokyo, Japan) on cassette tapes (D60 (Type I), TDK, Tokyo, Japan), using a directional condenser microphone (C 568 EB, AKG, Vienna, Austria) placed at ~100 cm in front of the focal male. The recordings were then digitized at a bit-depth of 16 bit and a sampling frequency of 44.1 kHz, using a laptop computer (PowerBook G3, Apple, Cupertino, CA, USA) and the sound processing software Canary 1.2.4 (Charif et al. 1995). Single call notes of the digitized recordings were then cut and re-aligned, using the acoustic software SoundEdit 2.0.7 (Macromedia; now Adobe, San José, CA, USA), to exhibit the average call properties of 15 males from the Arataï population as follows: number of notes per call, 4; note duration and frequency sweep range of note 1: 32.4 ms, 3011–3450 Hz; note 2: 66.1 ms, 2985–3846 Hz; note 3: 50.8 ms, 3004–3767 Hz; note 4: 64.0 ms, 3026–3932 Hz; inter-note intervals: notes 1 and 2: 50.2 ms; notes 2 and 3: 96.2 ms; notes 3 and 4: 43.9 ms; number of calls per bout: 10; inter-call interval (ICI): 458 ms; and inter-bout interval: 8.2 s.

**Fig. 1:**
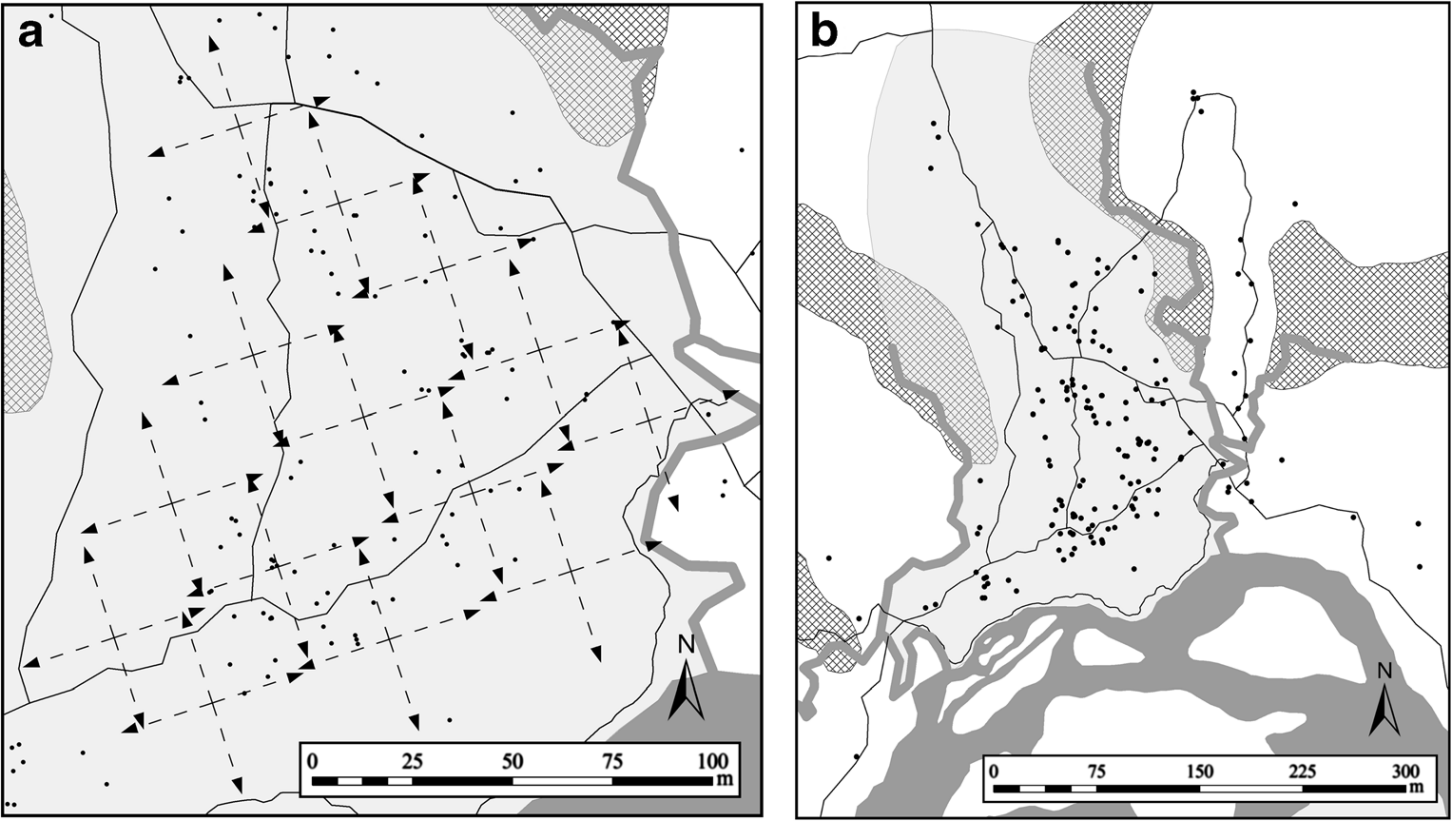
**a** Locations of the recording transects (*dashed arrows*) to obtain the test signals at 14 locations in the cardinal directions in the study plot (*light grey*). **b** Locations of 169 playback sessions (*black dots*) performed in the study population, situated in the study plot and adjacent areas. *Thin black lines* represent trails, *dark grey lines* show small creeks and the river Arataye, the cross-hatched area shows palm-swamps. The *north arrow* indicates geographic north, while the recording transects were laid out in relation to magnetic north (−18°) using a magnetic precision compass (Tandem, Suunto, Vantaa, Finland)

We broadcast and re-recorded this call in all four cardinal compass directions at 14 evenly spaced locations in the study plot (Fig. 1a) from 11 February to 11 March 2009 in the rainy season. At a few locations, one direction could not be recorded due to obstacles (large trees, river) along the recording transects, resulting in 52 unique recording sessions, yielding 312 unique signals. Transmission recordings were conducted between 1000 and 1400 h, the time of the lowest calling activity of *A. femoralis* males (Hödl 1983; Kaefer et al. 2012), to avoid interference with the recordings. All our recordings of naturally degraded *A. femoralis* calls were thus free from natural conspecific masking, i.e. contained no natural calls that were either audible or visible in the spectra. Additionally, the general background noise, emitted mainly by crickets and katydids, was lowest during this time period (MR pers. obs.; cf. Ellinger and Hödl (2003) for a rainforerst in Venezuela and Lang et al. (2005) for a rainforest in Panama).

The ‘standard call’, a 16-bit, 44.1-kHz WAV-file that contained a calling bout with 10 calls, was played using a portable audio player (G-Flash 512, Maxfield, Düsseldorf, Germany; company liquidated) and a portable battery-powered car-audio amplifier (Toxic TXC-500, RTO, Hamburg, Germany; maximum RMS power: 2 × 75 W, frequency range: 10–40,000 Hz) driving a portable full-range outdoor speaker (Symbol Pro 130, Magnat, Pulheim, Germany; frequency range: 35–30,000 Hz; high-frequency tweeter disabled for single membrane emission) with the speaker placed directly on the soil (centre of the membrane 9.25 cm above the soil, ~5 cm above the leaf litter). The sound reproduction system was calibrated before every broadcast, using a continuous, pure 400 Hz reference signal to produce an SPL of 95 dB (re 20 μPa; C, fast) at a distance of 0.75 m, resulting in an SPL of 97.8 dB (re 20 μPa; C, fast) of the *A. femoralis* test signal at this distance. The broadcast *A. femoralis* call was re-recorded simultaneously at six distances from the speaker. For recording, we used a portable outdoor computer (Toughbook CF-19, Panasonic, Osaka, Japan) and a USB-powered 6-channel audio A/D-interface (am6|2, Emagic, now Apple, Cupertino, CA, USA) with the audio recording software Audition 3.0 (Adobe, San José, CA, USA) at a bit-depth of 24 bit and a sampling frequency of 44.1 kHz. We used a directional microphone (ME66, Sennheiser, Wedemark-Wennebostel, Germany) at 0.75 m to record an immediate, unaltered far-field signal from the loudspeaker. At 1.5, 3, 6, 12 and 24 m, we used omnidirectional microphones (ME62, Sennheiser, Wedemark-Wennebostel, Germany) to capture the natural degradation as well as the reverberation signature of the signal when broadcast across the typical inter-individual communication range of *A. femoralis*. The microphones were mounted on small table-top tripods, ~10 cm above the soil, ~5 cm above the leaf litter and aligned horizontally, parallel to the forest floor, perpendicular to and pointing directly towards the membrane of the speaker. The position and vertical alignment of the speaker and microphones thus resembled the natural calling and listening positions of *A. femoralis* males, which call and listen on the leaf litter and from perches that are slightly elevated (10–20 cm; Hödl 1983).

We measured the signal-to-noise ratio (SNR) of the recordings using the ‘Inband Power (dB)’ (IP(dB)) measurement of the audio analysis software Raven Pro 1.5 (Bioacoustics Research Program 2011), which measures the sum of the square magnitudes of the Fourier coefficients in a selection, divided by the product of the DFT size and the number of spectrogram frames in the selection (Pitzrick 2016a), following the suggestions of the program’s developer (Pitzrick 2016b). Inband power was measured separately for each of the four notes of the first call in each recording and across the frequency range of the first formant of each note. The inband power of the background noise was measured from the 2-s period immediately before the first note, with separate measurements across the respective frequency range of each of the four following notes. All logarithmic measurements of inband power were then converted into linear (digital sampling) units (IP(u)) by using the formula *IP*(*u*) = 10^(*IP*(*dB*)/10)^. We then calculated a linear SNR(u) for each of the four notes using the formula *SNR*(*u*) = (*IP*(*u*)_*signal*_ − *IP*(*u*)_*noise*_)/*IP*(*u*)_*noise*_. We then calculated an SNR(u) for each recording by averaging the linear SNR(u) of each of the four notes and obtained an average SNR(u) for each distance by calculating the mean of all SNR(u)s for each distance. To obtain more commonly used logarithmic SNR measurements, we then reconverted SNR(u) to SNR(dB) using the formula *SNR*(*dB*) = 10 × log_10_ *SNR*(*u*).

**Fig. 2:**
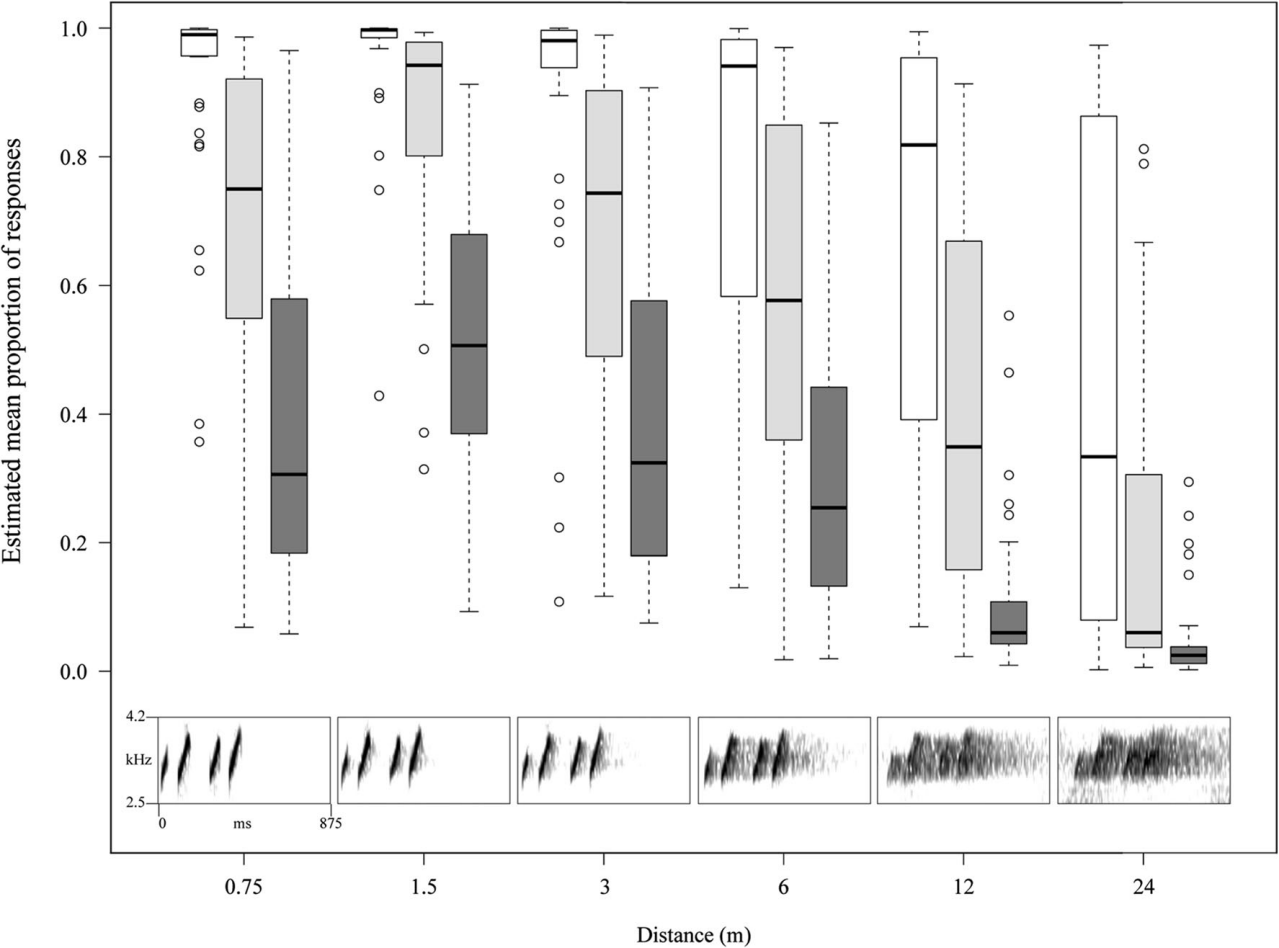
Estimates of mean proportions of HBO (*white*), movement (*light grey*) and approach (*dark grey*) responses (0 = no response; 1 = response) for different distances (distance_rec). Values are derived from the full models and are controlled for fixed and random effects. Shown are boxplots, with medians, quartiles, 1.5 × IQR and outliers beyond the 1.5 × IQR. *Insert* shows spectrograms of a test signal re-recorded across 0.75–24 m and normalized to 0 dbFS

To create the test signals for the playback trials with frogs, blocks of 8.8 s, containing the 10 calls and the reverberation after the last call, were cut from the recordings. The clips were high-pass filtered at 1.3 kHz and low-pass filtered at 12.0 kHz, to eliminate any noise below and above the *A. femoralis* call and its harmonics, and then amplitude-normalized to 0 dBFS (100%), using the (peak) normalization function of Adobe Audition (see insert in Fig. 2 for spectrograms). We looped the 8.2 s 10-call bouts and their 8.2 s lead-out (final reverberation plus 7.6 s silence) 10 times and added a silent lead-in of 3 s. Thus, a full test signal lasted for 2:47 min and presented 10 bouts of 10 calls, each consisting of 4 notes. We randomly ordered and stored all 312 unique test signals on a portable audio player for later use in the playback trials.

#### Playback trials

We performed playback trials with a portable audio player (Odys Smart 2 GB, Axdia International, Willich, Germany) driving a portable, amplified outdoor speaker with internal batteries (EcoxBT, Grace Digital, San Diego, CA, USA; maximum RMS power: 2 × 3 W; frequency range: 135–17,000 Hz, S/N-ratio: 88 dB ± 3 dB). We played the test signals from 24-bit, 44.1-kHz WAV-files, which were stored in randomized order on the audio player. We calibrated the playback setup twice per day on the forest floor to produce the undegraded ‘standard call’ with an SPL of 69 dB (re 20 μPa; A; fast) at a distance of 2 m, just above the threshold for phonotactic approaches in *A. femoralis* as reported by Hödl (1987).

For playbacks, we opportunistically approached calling *A. femoralis* males in the study area (‘mainland plot’ sensu Ringler et al. (2016)) and recorded the males’ location on a digital map on a portable GIS device (MobileMapper10, SpectraPrecision, Westminster, CO, USA). We carefully approached calling males and placed the speaker on the ground at ~2 m, pointing towards the focal male. The exact playback distance was measured with a rigid, foldable metre immediately after the trial. Playback directions were selected opportunistically to allow for unobstructed playback paths between focal frogs and the speaker and to give an unobstructed view to playback observers. After a resting period of at least 1 min after the speaker had been set up, we started the playback at times when the focal male, as well as any other immediate neighbouring males, were not calling. For the playback, we used the test signals consecutively in the random order as they had been stored in on the audio player. As *A. femoralis* males only show phonotaxis while a signal is present (Ursprung et al. 2009), it was not possible for us to follow the recommendations of Naguib and Wiley (2001) to play only short single signals to elicit a response in the focal individuals. Thus, we had to present the test signals throughout the trials.

During the playbacks, we commented behavioural observations using a small voice recorder (ICD-PX333, Sony, Tokyo, Japan). We recorded the following hierarchical phonotactic responses: ‘head-body-orientation’ (HBO), as soon as the frog oriented towards the speaker, followed by ‘movement’, as soon as the frog made the first jump towards the speaker, and eventually followed by a successful ‘approach’ within 20 cm of the speaker. We later transcribed and coded the recorded comments as binary responses for performed HBO, movement (following HBO), and approach (following HBO and movement). A trial was rated valid as soon as a frog showed at least HBO, and scored with all responses taking place until the end of the 2:47-min test signal. We stopped trials when the frog approached the speaker within 20 cm. When a frog showed no phonotactic response at all to a test signal, we immediately conducted a second trial without changing the speaker location by broadcasting the next random test signal and following the same trial protocol. If the frog showed a phonotactic response (at least HBO) to the second test signal, we scored a valid negative trial (no response) for the first test signal and a valid positive trial with the respective responses for the second test signal. If a frog also showed no response to the second test signal, we immediately conducted a third trial without changing the speaker location by broadcasting the next random test signal and following the same trial protocol. If the frog showed a phonotactic response (at least HBO) to the third test signal, we scored valid negative trials (no response) for the first and second test signal, and a valid positive trial with the respective responses for the third test signal. If a frog showed no phonotactic response to all three different random test signals, we immediately conducted a control playback, using the original, undegraded ‘standard call’, amplified by 6 dB, to verify the frogs’ general reactivity and territorial status. For frogs that showed a phonotactic response (at least HBO) to the control signal, we scored three valid negative trials for the three previous test signals, respectively. Frogs that also did not respond to the control signal were classified as non-territorial or unmotivated, and all three playback trials were discarded and not scored. When we observed another frog that was interacting physically or acoustically with the focal male, we stopped and discarded the trial and caught both individuals for registration. Frogs that had participated in a playback trial were not approached and tested again on the same day, but could be tested again in further trials on subsequent days.

After the trials, we caught the focal frog and took a digital picture (TG-620, Olympus, Tokyo, Japan) of the ventral pattern for identification, and a dorsal picture on mm-paper for the subsequent measurement of the snout-urostyle-length (SUL) in imageJ (Rasband 1997–2017). Then, we measured the playback distance between focal male and speaker, and the SPL (A; fast) of every signal used in this trial at the initial location of the focal male, using a sound pressure meter (Voltcraft SL-100, Conrad, Hirschau, Germany) to correct for the playback distance and the actually received SPL of the signal in the GLMM analysis. We also measured the ambient temperature and relative humidity at the location of the focal frog, using a thermo-hygrometer (GFTH 95, GHM Messtechnik, Regenstauf, Germany).

#### Sample size

In 175 playback sessions, we tested 117 different *A. femoralis* males. Because of equipment failures (interrupted or low playback sound due to lose contacts), we had to discard 6 sessions, leaving 169 sessions (Fig. 1b) in which 214 different test signals were used with 114 individuals. Of these males, 78 participated in one playback session, 21 twice, 11 three times and 4 four times. During these playback sessions, 68 males received 1 test signal, 17 received 2 different test signals, 17 received 3 different test signals, 6 received 4 different test signals, 2 received 5 different test signals, 2 received 6 different test signals, 1 received 7 different test signals and 1 received 8 different test signals. We aimed at performing at least 30 trials with each of the conditions (recording distance) of the test signals. Overall, we used 39 test signals that were recorded from 0.75 m, 41 test signals that were recorded from 1.5 m, 34 test signals that were recorded from 3 m, 37 test signals that were recorded from 6 m and 32 test signals that were recorded from 24 m. The exact number of test signals, their conditions and the breakdown of which male received which and how many test signals and during how many playback sessions is provided in the supplementary raw data.

Our unbalanced repeated measures design resulted from logistic constraints when conducting this study. Locating the frogs without disturbing them, setting up the playback equipment and catching the frogs and measuring environmental and trial parameters afterwards were considerably more time consuming than conducting the actual playbacks. Therefore, we aimed at allocating time available in the field most efficiently by using additional test signals with frogs that showed no response to the initial signals. We later accounted for this unbalanced design and repeated measurements on individuals with a varying number of test signals by integrating a corresponding random factor in our analysis.

#### Blinded methods

Playback trials were performed blindly to the extent that territorial males were approached opportunistically and their identity was only assessed after the trials; however, territoriality and site fidelity of the males provided information on male identity in the case of repeatedly tested individuals. The test signals were used in the randomized order they had been stored in on the music player previously, with the files specified only with a running number. However, to a certain degree, we could acoustically discern the test condition presented to the frog. Transcription and behavioural coding were done blindly, and acoustic characteristics of the test signals that would have allowed the test condition to be identified were barely audible in the voice-protocol recordings.

#### Statistics

To analyse the frogs’ responses to naturally degraded signals, we used generalized linear mixed models with the binary response variables ‘HBO’, ‘movement’ and ‘approach’. We included the signal characteristics ‘recording distance of the playback signal’ (distance_rec), ‘location recorded’ (location) and ‘direction recorded’ (direction) as fixed factors for our analysis. We included ‘day of trial’ (day), ‘time of trial’ (minute), ‘SPL’, ‘playback distance of the trial’ (distance_trial), ‘temperature during trial’ (temperature) and ‘humidity during trial’ (humidity) as characteristics of the playback trial. Finally, we used ‘snout-urostyle-length’ (SUL) as a physical characteristic of the tested frogs.

Variance inflation factors were calculated beforehand for all fixed factors in the model (distance_rec, location, direction, day, minute, SPL, distance _trial, temperature, humidity and SUL) to identify collinear parameters (Zuur et al. 2009), and ‘location’ and ‘day’ were excluded due to multicollinearity with other factors. A test of proportions was conducted to investigate whether the proportion of responses differed between recording locations and testing days. The proportions of responses did not vary with ‘location’ (HBO: *χ* ^*2*^ = 10.66, *df* = 13, *p* = 0.6393; movement: *χ* ^*2*^ = 10.514, *df* = 13, *p* = 0.6515; approach: *χ* ^*2*^ = 11.555, *df* = 13, *p* = 0.5644) or ‘day’ (HBO: *χ* ^*2*^ = 11.282, *df* = 11, *p* = 0.4199; movement: *χ* ^*2*^ = 8.3831, *df* = 11, *p* = 0.6786; approach: *χ* ^*2*^ = 6.5373, *df* = 11, *p* = 0.8352).

With the remaining factors, we fitted generalized linear mixed models for each response variable (HBO, movement, approach) using a binomial distribution with a logit link function within the lme4 package (Bates et al. 2016) in R (R Core Team 2017). A nested term including ‘signals received’ by each individual was used as random factor to account for repeated trials per individual. Starting with the full model that contained all fixed factors, a stepwise reduction was applied using likelihood ratio tests to determine whether the deletion of a factor from the model would significantly increase model fit. The full models (Table 1) did not vary significantly from the reduced models for either of the response variables (likelihood ratio tests: HBO: *χ* ^*2*^ = 4.452, *df* = 7, *p* = 0.7265; movement: *χ* ^*2*^ = 4.131, *df* = 4, *p* = 0.3886; approach: *χ* ^*2*^ = 2.113, *df* = 2, *p* = 0.3477), indicating that the full model had the best fit and explained variation in response variables best. Therefore, we present and discuss the results of the full model. To assess whether the probability of fully approaching the speaker up to 20 cm varied between the recording distances of the signals, we conducted *post hoc* Chi-square tests (Table 2). *p* values presented in Table 2 are two-tailed and alpha was set to 0.008 to account for multiple testing.

**Table 1:** Coefficients of the full generalized linear mixed-effect models with estimates

Coefficients	HBO (yes/no)				Movement (yes/no)				Approach (yes/no)			
	Estimate	SE	*z*	*p*	Estimate	SE	*z*	*p*	Estimate	SE	*z*	*P*
Intercept	18.623	25.314	0.736	0.4619	34.228	19.427	1.762	0.0781	24.046	16.540	1.454	0.1460
Distance_Rec (1.5 m)^a^	0.838	1.276	0.657	0.5114	1.420	0.797	1.781	0.0749	0.932	0.628	1.485	0.1375
Distance_Rec (3 m)^a^	−0.650	1.078	−0.603	0.5465	−0.119	0.733	−0.162	0.8714	0.189	0.633	0.299	0.7649
Distance_Rec (6 m)^a^	−1.178	1.018	−1.157	0.2473	−0.466	0.704	−0.661	0.5086	−0.445	0.633	−0.703	0.4822
Distance_Rec (12 m)^a^	−3.726	1.335	−2.791	**0.0053**	−2.118	0.871	−2.431	**0.0151**	−1.985	0.841	−2.361	**0.0182**
Distance_Rec (24 m)^a^	−4.798	1.459	−3.289	**0.0010**	−3.390	1.019	−3.326	**0.0009**	−2.871	1.021	−2.812	**0.0049**
SPL	0.265	0.137	1.943	0.0521	0.172	0.075	2.314	**0.0207**	0.205	0.066	3.122	**0.0018**
SUL	−0.704	0.430	−1.637	0.1016	−0.100	0.267	−0.373	0.7094	−0.013	0.221	−0.057	0.9546
Distance_Trial	0.027	0.021	1.305	0.1919	−0.032	0.016	−1.973	**0.0485**	−0.020	0.014	−1.357	0.1749
Minute	−3.333	3.384	−0.985	0.3247	−4.582	2.263	−2.025	**0.0429**	−3.407	1.876	−1.815	0.0695
Temperature	−0.377	0.385	−0.978	0.3279	−0.599	0.299	−2.000	**0.0455**	−0.339	0.248	−1.365	0.1723
Humidity	−0.028	0.175	−0.159	0.8739	−0.163	0.131	−1.248	0.2121	−0.231	0.119	−1.937	0.0527
Direction (N)^b^	−0.589	0.850	−0.693	0.4881	−0.117	0.600	−0.194	0.8459	0.221	0.563	0.392	0.6951
Direction (S)^b^	−0.732	1.000	−0.732	0.4639	0.819	0.660	1.243	0.2140	1.393	0.611	2.282	**0.0225**
Direction (W)^b^	0.138	0.844	0.164	0.8697	0.928	0.657	1.412	0.1579	0.811	0.581	1.395	0.1630

^a^For distance_rec, 0.75 m was set to zero

^b^For direction, east was set to zero

*SE* standard errors, *z* effect sizes and significance levels, *p* < 0.05 in bold

**Table 2:** χ^2^-tests of the probabilities to approach the speaker compared to chance, in response to distance-degraded signals

Distance (m)	Approach			
	*n* (yes, no)	Probability	*χ* ^*2*^	*p*
0.75	15.24	38.5%	1.641	0.2002
1.5	21.20	51.22%	0	1
3	13.21	38.24%	1.4412	0.2299
6	12.25	32.43%	3.8919	0.04852
12	4.27	12.9%	15.613	**>0.0001**
24	2.30	6.25%	22.781	**>0.0001**

Null-probablity = 0.5

Significant results after Bonferroni correction in bold

To investigate the influence of SNR on the frogs’ responses in the playback trials, we normalized the measured, linear SNR(u) values within each distance to a scale from 0 to 1 (SNR_norm). As the factor SNR_norm encoded the recording distance of the playback signals, this factor could not be tested in the same model that included recording distance. Thus, we calculated a separate model with a binomial distribution and a logit link function using a nested term that included the ‘signals received’ by each individual as a random factor and the normalized SNR(u) as a fixed factor.

### Experiment 2—playbacks with noise-masked signals

#### Test signals

For this experiment, we used the same ‘standard call’ as described for experiment 1 as a base signal. We used the audio editing software Audacity 2.1.2 (Audacity Team 1999–2017) to create two test conditions, masking the entire 10-call bout with broadband white noise with SNRs of 68.5 dB (high SNR) and 23.7 dB (low SNR) when measured in the frequency range of the *A. femoralis* standard call (2985–3932 Hz). These SNRs correspond to the average maximum and minimum SNR that we measured for the naturally degraded test signals in experiment 1 (Table 3) at the closest (0.75 m; high SNR) and furthest (24 m; low SNR) recording distance, respectively. The corresponding inter-bout interval of equal length (8.2 s) was filled with broadband white noise at the same amplitude as used to mask the respective signal. We looped both masked 10-bout signals and their noisy interbout-intervals 10 times and added a 3-s lead-in to obtain the two test signals with a length of 2:47 min.

**Table 3:** Mean signal-to-noise ratios of the recorded signals, calculated from SNR (u)

Distance (m)	Mean SNR (dB)	Range (dB)	Mean SNR (u)	SD SNR(u)
0.75	68.5	21.9	7,094,206	7,148,242
1.5	55.5	25.6	355,896	563,389
3	44.8	26	30,431	43,465
6	35.6	15.8	3633	3302
12	30.3	20.3	1070	937
24	23.7	16.4	237	196

#### Playback trials

To conduct the playback trials, we used the same equipment, daily calibration protocol (unmasked signal at 2 m, 69 db (re 20 μPa; A; fast)) and spatial setup as described for experiment 1, but we placed the speaker at exactly 2 m from the focal individual, using a 2 m PVC tube. We tested the phonotactic responses of 16 *A. femoralis* males to the high SNR and low SNR test signal in a pairwise design, where we alternately used either condition in the first trial on the tested males. Males were never tested twice on the same day, and the respective other test condition was used in a second playback trial on subsequent days. We played the entire 2:47 min test signals to the focal individual and recorded the same hierarchical phonotactic responses as in experiment 1. After the trials, we captured the frogs, took ventral photographs for identification, registered the location on a digital map and measured the ambient temperature and humidity. As humidity was at 100% throughout all trials, we discarded this environmental parameter in the further analysis. To account for variation in signal transmission in the natural environment and potential intra-day variation in speaker output, after a playback trial, we measured the SPL (A; fast) of the non-masked original ‘standard call’ at the initial location of the focal frog.

#### Blinded methods

The playback experiments were not conducted blindly, as we used a paired design and alternated the test condition in the first trials with each individual, respectively. However, for the first trials, calling, territorial focal males were approached and tested opportunistically in the order that they were detected in the study area. Coding of the behavioural recordings was also not done blindly, as the signal condition (high SNR/low SNR) was clearly audible on the voice recordings.

#### Statistics

As all tested frogs responded to both playback stimuli by orienting and moving towards the speaker, we only fitted a generalized linear model for the response variable ‘approach’. We included signal condition (high SNR–low SNR), SPL (of the non-masked original ‘standard call’ at the initial location of the focal frog), day of trial and temperature during trial as fixed factors. Day of trial was removed from the analysis due to collinearity with temperature. A test of proportions revealed no significant differences in the proportions of responses between testing days (*χ* ^*2*^ = 5.3333, *df* = 7, *p* = 0.6194). The remaining fixed factors (signal condition, SPL and temperature) were used to fit a generalized linear model with a binomial distribution and a logit link function. As in the previous models, a stepwise reduction of the full model did not result in a significant increase in model fit (likelihood-ratio test: *χ* ^*2*^ = 0.194, *df* = 2, *p* = 0.9076), which is why we report and discuss the full model.

## Results

### Experiment 1—playbacks with degraded signals

#### General results

In the 169 playback trials, the playback distance varied between 140 and 260 cm, and post-trial measured playback SPL varied between 54.5 and 73.5 dB. Between 0905 and 1940 h, during the time when the playbacks were performed, the temperature ranged between 21.5 and 29.5 °C and relative humidity varied from 83 to 100%. Males showed positive HBO towards 165 of the 214 different test signals, movement was elicited in 123 cases, and 67 signals led to a successful approach to the speaker. The full information on responses to specific test conditions is provided with the supplementary raw data.

#### Responses to playbacks

The recording distance of the playback signal (distance_rec) had a significant effect on the frogs’ probability to orient towards, move towards and successfully approach the speaker (see Table 1). Responses were highest for close distances and decreased for longer distances (Fig. 2). *Post hoc* analysis revealed that the probability to fully approach the speaker up to 20 cm did not deviate from chance level up to 6 m but significantly decreased below chance level for distances beyond 6 m (Table 2).

**Fig. 3:**
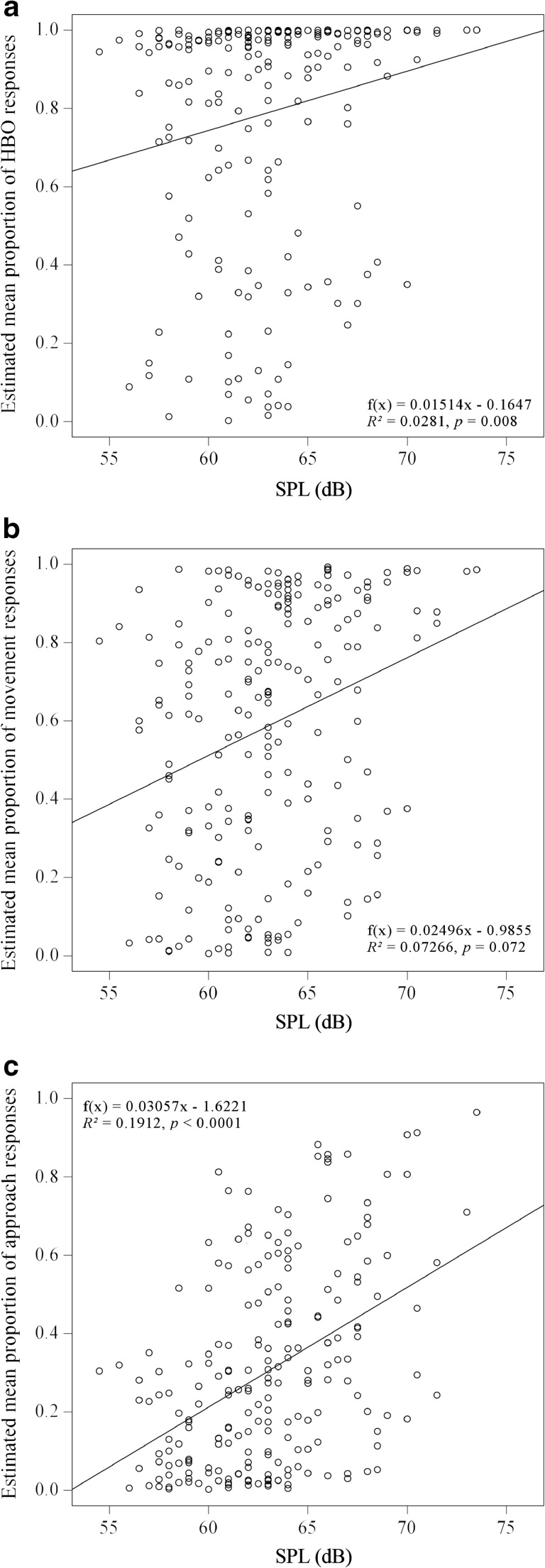
Estimates of mean proportions of **a** HBO (f(x) = 0.01514x–0.16472; 50% response probability at 43.9 dB), **b** movement (f(x) = 0.02496x–0.9855; 50% response probability at 59.52 dB) and **c** approach (f(x) = 0.03057x–1,6221; 50% response probability at 69.42 dB)) responses for different SPLs. Values are derived from the full models and are controlled for fixed and random effects

SPL significantly influenced movement (estimate ± SE = 0.172 ± 0.075; *z* = 2.314; *p* = 0.0207) and approach responses (estimate ± SE = 0.205 ± 0.066; *z* = 3.122; *p* = 0.0018) with higher proportions of responses for higher SPLs. The influence of SPL on the proportion of HBOs was marginally significant (estimate ± SE = 0.265 ± 0.137; *z* = 1.943; *p* = 0.0521; Fig. 3).

In addition to the recording distance of the playback signal and the SPL, movement towards the loudspeaker was also significantly influenced by distance over which a playback trial was conducted (distance_trial; estimate ± SE = −0.032 ± 0.016; *z* = −1.973; *p* = 0.0485), minute (estimate ± SE = −4.582 ± 2.263; *z* = −2.025; *p* = 0.0429) and temperature (estimate ± SE = −0.599 ± 0.299; *z* = −2.0; *p* = 0.0455), with decreasing probabilities to respond when the playback distance, minute and temperature increased. Approaches towards the speaker were marginally affected by the humidity (estimate ± SE = −0.231 ± 0.119; *z* = −1.937; *p* = 0.0527), with slightly lower probabilities to approach with increasing humidity and direction in addition to the recording distance of the signal and SPL (Table 1).

#### Influence of SNR

**Fig. 4:**
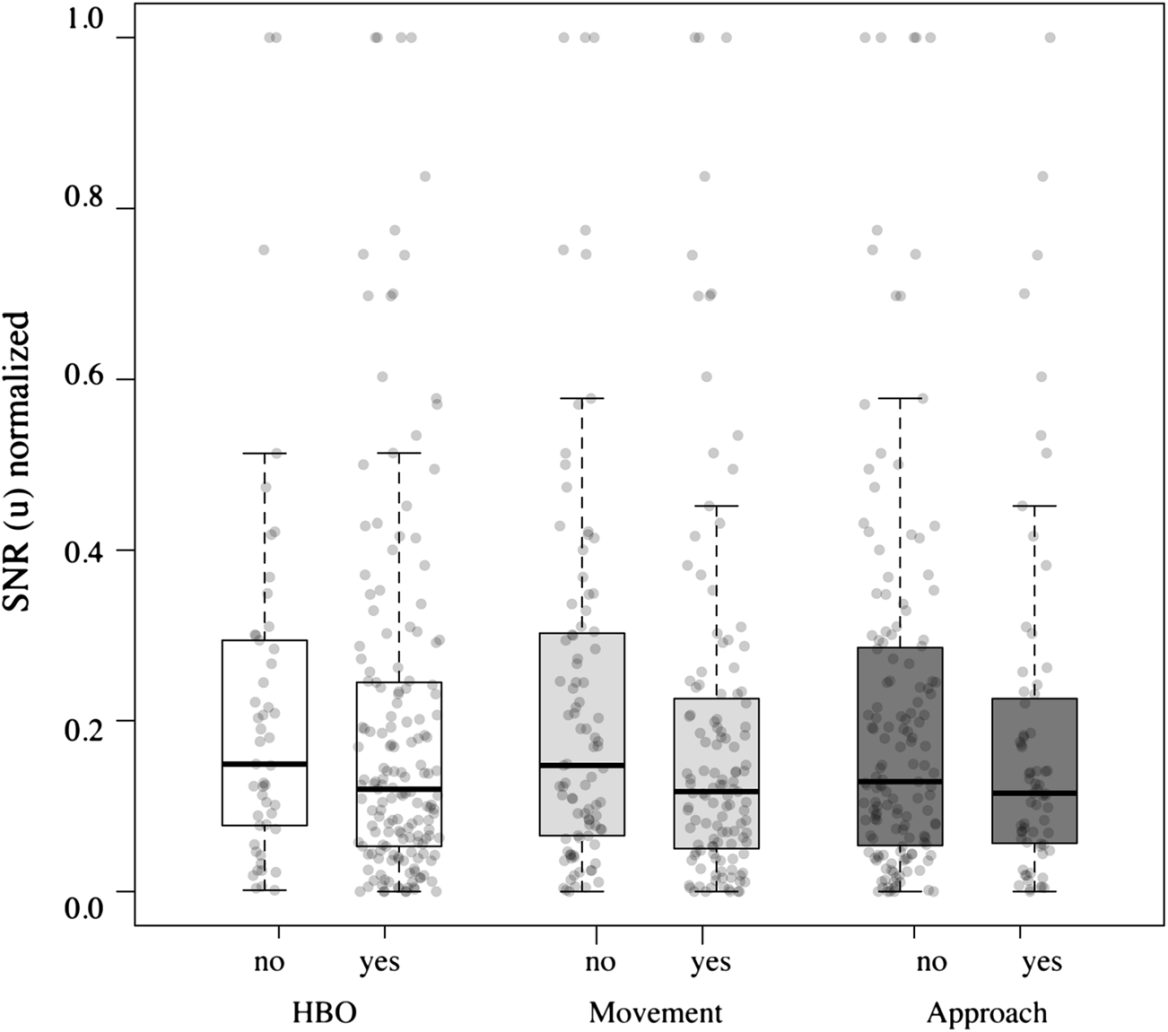
Box plots with medians, quartiles and 1.5 × IQR of the normalized SNR (u) measurements of the test signals from trials where frogs responded (‘yes’) or did not respond (‘no’) by HBO, movement or full approach. *Dots* indicate SNR (u) values of single recordings and are jittered horizontally, proportional to the sample size in each category

The mean SNR values of the test signals within each of the six distances ranged from 23.7 to 68.5 dB, well above any critical SNR threshold reported for frogs (cf. Vélez and Bee 2010, 2011; Velez et al. 2013; Wiley 2015), and showed a natural variation between 16.3 and 26 dB within each distance (Table 3). The SNR of the signals, normalized within each signal recording distance, had no significant effect on any of the frogs’ responses (Table 4, Fig. 4).

**Table 4:** Coefficients of the linear models to assess the effect of SNR with estimates

	Coefficients	Estimate	SE	*z*	*p*
HBO (yes/no)	Intercept	2.197	0.544	4.041	**<0.0001**
	SNR_norm	−0.589	0.903	−0.652	0.514
Movement (yes/no)	Intercept	0.856	0.305	2.807	**0.005**
	SNR_norm	−1.195	0.784	−1.524	0.127
Approach (yes/no)	Intercept	−0.661	0.227	−2.912	**0.003**
	SNR_norm	−0.656	0.771	−0.850	0.395

*SE* standard errors, *z* effect sizes and significance levels, *p* < 0.05 in bold

### Experiment 2—playback with masked signals

#### Responses to playbacks

In four out of 32 playbacks, frogs did not respond by approaching the speaker. However, the frogs’ decision to approach the speaker was not influenced by the signal condition (high or low SNR; estimate ± SE = 0.516 ± 1.260; *z* = 0.409; *p* = 0.6822) nor did the SPL (estimate ± SE = −0.578 ± 0.333; *z* = −1.736; *p* = 0.0826) or temperature (estimate ± SE = −0.016 ± 0.699; *z* = −0.024; *p* = 0.9812) affect responses (Table 5).

**Table 5:** Coefficients of the full generalized linear model investigating the effects of high and low SNR on approaching responses

Coefficients	Estimate	SE	*z*	*p*
Intercept	41.542	28.626	1.451	0.1467
Condition (low/high)	0.516	1.260	0.409	0.6822
SPL	−0.578	0.333	−1.736	0.0826
Temperature	−0.016	0.699	−0.024	0.9812

Estimates with standard errors (SE), effect sizes (z) and significance levels are shown

**Fig. 5:**
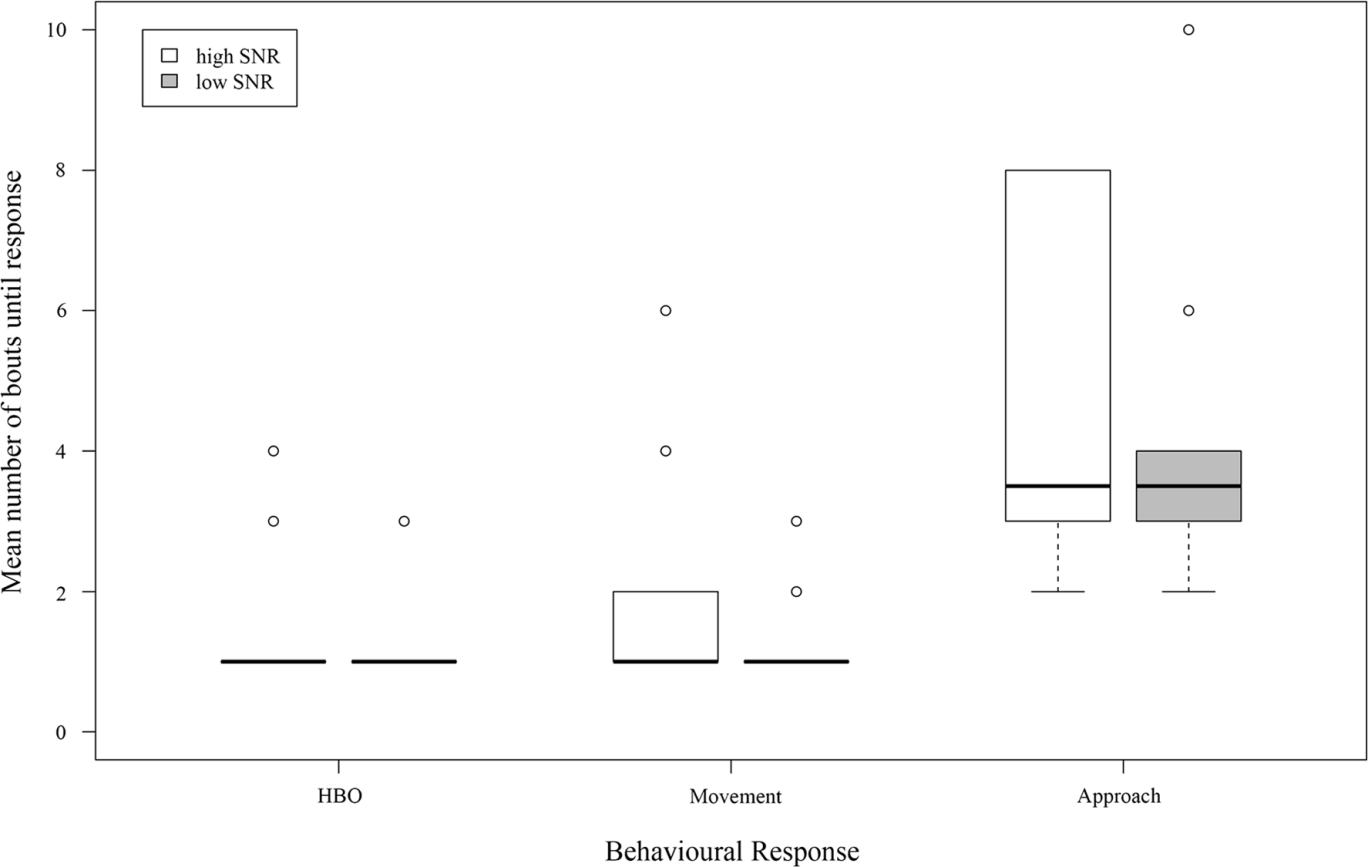
Box plots with medians, quartiles, 1.5 × IQR and outliers beyond the 1.5 × IQR, showing the number of bouts until a frog showed an HBO, movement or approach response to conspecific ‘standard calls’ with a high SNR (*left boxes*; *white*) or low SNR (*right boxes*; *grey*)

The number of the bout (1–10) when a phonotactic response was shown by the tested frog did not differ between stimuli with high and low SNR for any of the response variables (Wilcoxon signed-rank test: HBO: *n* = 16, Z = 1.5, *p* = 0.5862; movement: *n* = 16, Z = 3.0, *p* = 0.2785; approach: *n* = 14, Z = 24.0, *p* = 0.2505; Fig. 5).

## Discussion

### Cognitive aspects

Independently of the actual SPL and SNR of the signals, the tested *A. femoralis* males showed differential HBO, movement and approach responses in our playback trials with naturally degraded signals, depending on the recording distances of broadcast conspecific advertisement calls. In our follow-up playback experiment using signals that were masked with high- and low-amplitude noise, the signal-to-noise ratio had no discernible effect on the frogs’ propensity to orientate to, move towards, and fully approach the loudspeaker. These results clearly indicate that the frogs use SPL and SNR only as ancillary distance cues when responding to territory intruders that were simulated at varying distances and posing different levels of threat. Nevertheless, consistent with the previously reported threshold for phonotaxis in *A. femoralis* (Hödl 1987), signal SPL also influenced the responses of frogs tested in our trials. The previously reported response thresholds for a Peruvian *A. femoralis* population, 56–68 dB for HBO and >68 dB for phonotactic movement correspond well with our observations. These thresholds result in probabilities of 68–86% for an HBO, 71% for initiated movement and 45% for a successful approach in our study (cf. regression functions in Fig. 3a).

The differential responses to near (0.75–6 m) and far signals (12–24 m) suggest a threshold distance for aggressive responses to nearby callers. The cut-off between 6 and 12 m corresponds to the average distance of phonotactic approach (7.07 ± SD 3.5 m) and the area defended in a previous study, using systematic playback experiments (‘playback territories’ = 151.13 m^2^; radius of equi-areal circle = 6.94 m; Ringler et al. 2011). While the difference in responses to near and far signals was most pronounced for full approaches to the playback speaker (Fig. 2; Table 1), initial orientation and commenced movement towards the playback speaker were also affected by the signals’ recording distance (Table 1). Because both hierarchical responses, HBO and initiation of movement, took place at the initial location of the tested frogs, they could not have used additional locational information such as SPL-gradients, change in signal degradation, or triangulation while approaching the speaker (cf. Murphy 2008). However, we cannot rule out that such effects influenced the differential rate of full phonotactic approaches to near and far signals.

In experiment 2, frogs responded equally and with equal latency in all three response categories in playbacks with noise-masked signals, featuring the average maximum and minimum SNR of the naturally degraded signals. This indicates that SNR did not influence phonotactic behaviour in these trials and plays no prominent role in distance assessment across the typical inter-individual distances of *A. femoralis*. All tested frogs orientated and moved towards playback signals with low and high SNR, and only 2 out of 16 frogs did not eventually reach the speaker in either condition. This corroborates previous findings by Hödl et al. (2004) who showed that even masking noise with an SPL equal to the SPL of the pure signal allowed for at least some signal recognition, eliciting HBO and movement in 12.5% of the cases. Notably, our test signals had been recorded under natural conditions but at times without conspecific calling activity. Conspecific calling is known to have the strongest masking effect, inhibiting call recognition and detection (Wollerman 1999; Wollerman and Wiley 2002). Frogs also paid rather high attention (HBO) to more degraded signals in experiment 1 and responded indifferent to low and high SNR’s in experiment 2. This is in line with the previously identified rather broad temporal recognition space of *A. femoralis* (Göd et al. 2007; Amézquita et al. 2011; Vélez et al. 2012; Betancourth-Cundar et al. 2016). Hence, we conclude that signal detection and recognition were not jeopardized under the conditions of experiment 1 when using normalized, naturally degraded signals (cf. Naguib and Wiley 2001; Venator et al. 2017). The masking noise in the test signals in experiment 2, featuring minimum and maximum SNRs of experiment 1 but way below any critical threshold for frogs (cf. Vélez and Bee 2010, 2011; Velez et al. 2013; Wiley 2015), was sustained during the inter-bout intervals and its level did not affect the frogs’ phonotactic behaviour. Therefore, we also rule out any aversive effects of amplified background noise stemming from the normalization of the naturally degraded signals to obtain equal amplitudes, in experiment 1.

### Behavioural ecology

Dendrobatid frogs are renowned for their pronounced territoriality (Pröhl 2005; Lötters et al. 2007), exhibited via prominent acoustic displays and physical aggression. Distance assessment, as demonstrated in this study, probably plays a prominent role in establishing and maintaining stable territorial systems in poison frogs (cf. Narins et al. 2003; Gardner and Graves 2005; Ringler et al. 2009, 2011). As for most frogs, acoustic communication also is important in mate choice for dendrobatids, with females using male territoriality and calling to make mating decisions (Roithmair 1992, 1994; Pröhl 2003; Ursprung et al. 2011; Meuche et al. 2012, 2013; Ringler et al. 2012). It has been speculated that female *A. femoralis* monitor the calling activity of males that have sired clutches with these females to be able to compensate for when a mate becomes unavailable to perform tadpole transport (Ringler et al. 2015a). Accurate assessment of the distance of calling males, in combination with their direction and probably identity, would improve such monitoring by the females. Thus, female poison frogs would also benefit from elaborate distance assessment, assuming that they possess the same abilities as males.

*Allobates femoralis* has previously been shown to possess remarkable homing and orientation abilities (Pašukonis et al. 2013, 2014a), relying on spatial learning and memory (Pašukonis et al. 2014b, 2016), which has also been shown in another dendrobatid (Liu et al. 2016). As previously suggested for *A. femoralis* (Pašukonis et al. 2014a), we speculate that in this context, accurate ranging in combination with signal direction (cf. Ursprung et al. 2009) and caller identity (Gasser et al. 2009) could be used to establish a cognitive acoustic map (van Hemmen 2002; Gunina 2011; Fagan et al. 2013) of the local area. This ability has been found also in some birds (e.g. Hagstrum 2013) and mammals (e.g. Briseño-Jaramillo et al. 2015) that integrate acoustic information to memorize the position and identity of individual callers. This ability would be particularly useful in the context of tadpole transport, when males and, in rare occasions, females (Ringler et al. 2015a) have to navigate in their habitat to reach widely dispersed aquatic rearing sites for their offspring (Ringler et al. 2013; Pašukonis et al. 2014b, 2016; Erich et al. 2015).

### Experimental considerations

For playback experiments on ranging, it is generally recommended to avoid any close-range experience of the test subjects with the signal source, and no additional direct cues should be given during the approach of the animals. On the one hand, this protocol should preclude the animal from gathering further information that becomes available while approaching the sound source (SPL-gradient, triangulation, change in degradation), as further decisions on sustained movement and an eventual full approach could be made later, when more information becomes available at closer distances to the sound source. On the other hand, positive phonotaxis to a single distant stimulus is interpreted as a reliable indicator of signal detection and recognition of the degraded signal (Naguib 1996; Naguib and Wiley 2001). For *A. femoralis*, the territorial setup and typical inter-individual distances (Ringler et al. 2011), the general phonotactic SPL response thresholds (Hödl 1987) and the strong tendency to only move during signal perception (Ursprung et al. 2009) currently preclude such an experimental setup. Future studies will have to elucidate further whether frogs do have a cognitive representation of acoustic distance, comparable to birds and mammals, or whether the differential responses in our trials actually resulted from a continuous, graded response to the continuous, graded stimulus of our naturally degraded test signals. However, such experiments will have to bypass the behavioural and experimental limitation that frogs only show positive phonotaxis in the presence of a stimulus (Ursprung et al. 2009). Thus, unlike similar experiments in birds, no ‘overshooting’ or ‘undershooting’ of the speaker towards far and near signals, respectively, would be expected as a response towards an initial, triggering signal (Naguib 1996; Naguib and Wiley 2001). However, unlike birds, frogs have the advantage that they can generally be observed during their entire phonotactic responses, owing to the relatively smaller distances involved. Future studies should consider this advantage when planning the experimental setup.

In our present study, we were not yet able to identify the exact cues used for ranging by *A. femoralis*, as several signal characteristics are known to predictably co-vary with transmission distance. Such cues are temporal degradation, frequency-dependent excess attenuation and reverberation signature, which in our test signals were all collinear, given our experimental protocol of normalizing naturally degraded signals. Thus, it will require further playback experiments with systematic modification of single cues to elucidate the exact ranging mechanisms in *A. femoralis*.

## Data accessibility

All transcriptions of the playback trials and the corresponding environmental and individual frogs’ parameters are available as electronic supplementary material.

## Electronic supplementary material

ESM 1 (TXT 21 kb).

ESM 2 (TXT 2 kb).
